# Systemic lymph node tuberculosis presenting with an aseptic psoas abscess caused by a paradoxical reaction after nine months of antituberculosis treatment: a case report

**DOI:** 10.1186/1752-1947-7-72

**Published:** 2013-03-14

**Authors:** Gen Yamada, Hirotaka Nishikiori, Masaru Fujii, Shin-ichiro Inomata, Hirofumi Chiba, Naoki Hirokawa, Hiroki Takahashi

**Affiliations:** 1 The Third Department of Internal Medicine, Sapporo Medical University School of Medicine, Chuo-ku, South 1 West 16, Sapporo, 060-8543, Japan; 2 The Department of Radiology, Sapporo Medical University School of Medicine, Chuo-ku, South 1 West 16, Sapporo, 060-8543, Japan

## Abstract

**Introduction:**

A paradoxical reaction during antituberculosis treatment is defined as the worsening of pre-existing tuberculosis lesions or the appearance of a new tuberculosis lesion in patients whose clinical symptoms improved with antituberculosis treatment. The median onset time to the development of a paradoxical response has been reported to be about 60 days after the start of treatment. We report the case of a patient with a paradoxical reaction presenting as a psoas abscess after nine months of antituberculosis treatment. To the best of our knowledge, this manifestation has not previously been reported.

**Case presentation:**

A 23-year-old Japanese man presented to our hospital with lower abdominal pain. Computed tomography showed that he had mediastinal and abdominal para-aortic lymph node swellings. Fluorine-18 fluorodeoxyglucose positron emission tomography showed hot spots in these lymph nodes and in his right cervical lymph node, suggesting a lymphoma. The examination of an abdominal lymph node biopsy specimen showed lymph node tuberculosis, so antituberculosis treatment was started. However, after nine months of treatment, he experienced right flank pain. Abdominal computed tomography showed a right psoas abscess and abdominal para-aortic lymph node swelling. The abscess was treated by percutaneous drainage. After repeated drainage, the psoas abscess subsided and disappeared. The purulent fluid yielded no microorganisms, suggesting a paradoxical reaction.

**Conclusion:**

Attention should be paid to paradoxical reactions occurring during antituberculosis treatment for systemic lymph node tuberculosis.

## Introduction

A paradoxical reaction during antituberculosis (TB) treatment is described as a relatively rare manifestation and defined as the clinical or radiological worsening of pre-existing TB lesions or the development of new lesions in a patient who initially improves [1-3]. It affects 6% to 30% of patients receiving anti-TB treatment. The median time to development of a paradoxical reaction has been reported to be about 60 days after the start of treatment [1]. Diagnosis is made after the exclusion of other causes, such as secondary infection, inadequate treatment, drug resistance, poor compliance or drug adverse effects.

Psoas abscesses are usually the result of an infectious process, and are classified into primary or secondary abscesses [4]. A primary psoas abscess is spread via the bloodstream from a distant site in the body. A secondary psoas abscess is a result of the direct spread of an infection to the psoas muscle from adjacent structures such as the vertebral bodies and discs, the gastrointestinal tract, the genitourinary tract, and other sites. The infections in these organs are able to spread contiguously to the psoas muscle [5].

We report the case of a patient with systemic lymph node TB presenting with the paradoxical reaction of an aseptic psoas abscess that appeared after approximately nine months of anti-TB treatment. Computed tomography (CT)-guided percutaneous drainage of the abscess fluid yielded no microorganisms, suggesting a paradoxical reaction. To the best of our knowledge, a paradoxical reaction presenting as an aseptic psoas abscess after a sufficient course of anti-TB treatment has not previously been reported.

## Case presentation

**Figure 1:**
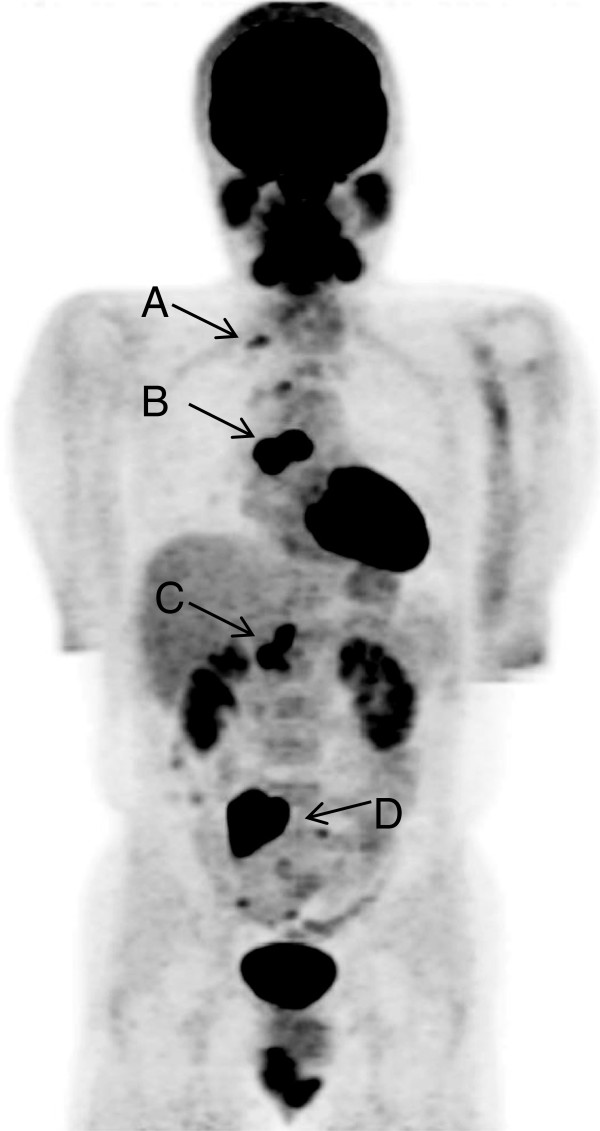
**Fluorine-18 fluorodeoxyglucose positron emission tomography findings.** Arrows show hot spots in cervical (**A**, standardized uptake value 2.8), mediastinal (**B**, standardized uptake value 15.9), abdominal (**C**, standardized uptake value 6.8) and common iliac (**D**, standardized uptake value 15.8) lymph nodes, suggesting a lymphoma.

A 23-year-old Japanese man presented to our hospital with lower abdominal pain. He showed no symptoms such as fever, weight loss, night sweats or anorexia. He did not smoke cigarettes and had not had any contact with patients with TB. His prior history was unremarkable and a physical examination was normal. His superficial lymph nodes were not palpable. The results of common blood cell counts and blood chemistry were within normal limits. His erythrocyte sedimentation rate was 36mm/h and his serum level of C-reactive protein was 0.95mg/dL (normal range: 0 to 0.3mg/dL). A QuantiFERON-TB (Cellestis International Pty Ltd., Chadstone, Victoria, Australia) test was positive. Examination of his sputum revealed no atypical cells and no microorganisms. A chest radiograph showed no abnormal findings. Chest and abdominal CT revealed enlarged mediastinal and abdominal para-aortic lymph nodes. Fluorine-18 fluorodeoxyglucose positron emission tomography showed hot spots in his right cervical, mediastinal and para-aortic lymph nodes, suggesting a lymphoma (Figure 1).

**Figure 2:**
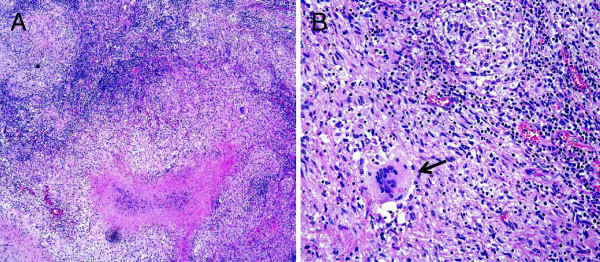
**Pathological findings: the biopsy specimen of the para-aortic lymph node shows necrotizing lymphadenitis with epithelioid cell granulomas.** (**A**) Magnification ×100. (**B**) Magnification ×400. Arrow shows multinucleated giant cells. (Hematoxylin and eosin stain staining).

A laparotomy was performed to obtain a sample to make a pathological diagnosis of his lymph node swelling. The biopsy specimen showed necrotizing lymphadenitis with epithelioid cell granulomas, which was consistent with *Mycobacterium* infection (Figure 2). Acid-fast bacteria were not found in either staining or culture of the biopsy specimen, and polymerase chain reaction (PCR) tests for *Mycobacterium tuberculosis*, *M*. *avium* and *M*. *intracellulare* were negative. Our patient was treated for lymph node TB with rifampicin 300mg, isoniazid 300mg, pyrazinamide 1200mg and ethambutol 750mg once a day. Two months later, his regimen was modified to rifampicin, isoniazid and ethambutol.

Three months after the start of treatment, our patient’s right supraclavicular lymph node appeared enlarged and he complained of low grade fever that lasted for about a month. A PCR test of aspirated fluid from his lymph node was positive for *M*. *tuberculosis*. However, acid-fast staining was negative and *M*. *tuberculosis* was not cultured from the fluid. Six months after the start of treatment, the enlargement of his supraclavicular lymph node improved and the anti-TB regimen was reduced to isoniazid and rifampicin.

**Figure 3:**
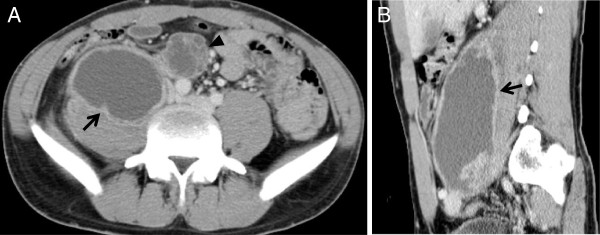
**Contrast-enhanced computed tomography findings.** Abdominal computed tomography shows a low-attenuation mass (arrow) with enhancing rim in the right psoas muscle. A para-aortic lymph node (arrowhead) adjacent to the psoas muscle is enlarged. (**A**) Axial view; (**B**) sagittal view.

Nine months after the start of treatment, our patient complained of right flank pain with radiation to his right hip and right thigh. A neurological examination was normal. Common blood cell counts and urine analysis were normal. His serum level of C-reactive protein was 0.95mg/mL. An abdominal CT revealed a large low-attenuation mass in his right psoas muscle and an enlarged para-aortic lymph node adjacent to the muscle (Figure 3). No enlargement of any other lymph node was found. The size of the abscess was 7.0cm×6.0cm×15.0cm and his para-aortic lymph node was approximately 3.0cm in diameter. CT-guided percutaneous drainage of the psoas abscess was performed, obtaining 280mL of a dense, purulent fluid. Acid-fast bacteria staining of the fluid was negative and PCR tests for *M*. *tuberculosis*, *M*. *avium* and *M*. *intracellulare* were negative. No bacteria or fungi were isolated from the fluid. The level of adenosine deaminase in the fluid was elevated to 245IU/L. After drainage, his right flank pain improved. However, the pain recurred a month later, and an abdominal CT showed that the abscess had relapsed. Drainage was performed again, obtaining 210mL of fluid of a similar consistency.

After repeated drainage, the psoas abscess subsided to 4.5cm×3.0cm×9.0cm and the lymph node to 1.5cm in diameter. As a result, anti-TB drugs were continued for 13 months. Two months after the end of treatment, the disappearance of both the abscess and the lymph node swelling were confirmed by abdominal CT. Our patient improved and his post-treatment period was uneventful. No sign of relapse had been detected 15 months after the end of treatment.

## Discussion

We have described the unusual presentation of a paradoxical reaction as an aseptic psoas abscess, which occurred late after starting treatment. A paradoxical reaction during treatment for lymph node TB may not present only as lymph node enlargement. This case is interesting because of the presentation of a paradoxical reaction as an extranodal disease and the late presentation of the reaction.

A microbial diagnosis is achieved in most patients with a psoas abscess through cultures of drained samples from the abscess. *M*. *tuberculosis* has been reported as the fourth most common organism, accounting for 7.5% of iliopsoas abscesses with a definitive microbial diagnosis [6]. In our case, the collected purulent fluid drained from the abscess yielded no microorganism and a PCR test for *M*. *tuberculosis* was negative. However, we considered TB as the cause of the aseptic psoas abscess because the para-aortic lymph node adjacent to the psoas muscle was enlarged compared with the initial imaging. A paradoxical reaction was suggested in the pathogenesis of the abscess during anti-TB treatment.

Paradoxical reactions usually occur relatively early after the start of combination chemotherapy. A possible explanation for the pathogenesis is thought to be related to the prompt recovery of the immune system to TB antigens after the use of anti-TB drugs. The pathogenesis was similar to immune reconstitution syndrome reported in patients with human immunodeficiency virus-related TB [7]. Our patient was immune competent and we believe that his immune reaction to *M*. *tuberculosis* was suppressed by systemic lymph node TB at the onset of infection. His psoas abscess may have been caused by contiguous spread from his para-aortic lymph node TB, followed by a paradoxical reaction. We suggest that his immune reaction to *M*. *tuberculosis* gradually recovered during the treatment for TB and caused an excessive reaction to *M*. *tuberculosis*, resulting in the paradoxical reaction. In addition, his supraclavicular lymph node enlargement might also have been a paradoxical reaction, as it appeared three months after starting treatment, which is compatible with a paradoxical reaction.

Treatment of paradoxical reaction includes surgical intervention and administration of steroids [1]. However, a paradoxical reaction is usually a transient and self-limited response in immune-competent patients. The management of a paradoxical reaction is the continuation of anti-TB treatment without any modification of the anti-TB drugs [8]. In our case, we continued anti-TB treatment and performed CT-guided percutaneous draining of the abscess [9]. The percutaneous catheter drainage was an effective alternative to surgical drainage as a supplement to medical therapy in the management of a psoas abscess.

Several risk factors for paradoxical reactions in patients without human immunodeficiency virus have been reported. Cheng *et al*. [3] found that baseline anemia, hypoalbuminemia, lymphopenia and a greater change in lymphocyte count were independent risk factors for the development of a paradoxical reaction. Cho *et al*. [10] found that younger age, male gender and the presence of local tenderness were associated with paradoxical reaction in lymph node TB, similar to our case.

Throughout the clinical course, no swelling was found in other lymph nodes except the abdominal para-aortic and the supraclavicular lymph nodes. It is unclear why paradoxical reactions after treatment are different in different sites of the body. The burden of a large amount of *M*. *tuberculosis* in lymph nodes or extranodal sites at the onset of TB might be related to paradoxical reactions.

## Conclusion

It is important for clinicians to recognize paradoxical reactions during anti-TB treatment. Our patient showed an unusual manifestation of aseptic psoas abscess, which occurred late after starting treatment. Paradoxical reactions during anti-TB treatment for systemic lymph node TB may not manifest only as lymph node enlargement; extranodal diseases should also be considered.

## Consent

Written informed consent was obtained from the patient for publication of this case report and accompanying images. A copy of the written consent is available for review by the Editor-in-Chief of this journal.

## Abbreviations

CT: Computed tomography; PCR: Polymerase chain reaction; TB: Tuberculosis.

## Competing interests

The authors declare that they have no competing interests.

## Authors’ contributions

HN, MF, SI, HC and HT analyzed and interpreted the patient data regarding the lymph node tuberculosis with psoas abscess. NH performed the CT-guided percutaneous drainage of the abscess. GY was a major contributor in writing the manuscript. All authors read and approved the final manuscript.
